# Recent population history in Swedish cattle breeds

**DOI:** 10.1186/s12711-026-01050-z

**Published:** 2026-05-24

**Authors:** Dolapo Adepoju, J. Ingemar Ohlsson, Tomas Klingström, Elisenda Rius-Vilarrasa, Anna M. Johansson, Martin Johnsson

**Affiliations:** 1 Department of Animal Biosciences, Swedish University of Agricultural Sciences, Box 7023, 750 07 Uppsala, Sweden; 2 Växa Sverige, 104 25 Stockholm, Sweden; 3 Beijer Laboratory for Animal Science, Swedish University of Agricultural Sciences, Box 7024, 750 07 Uppsala, Sweden; 4 Present Address: Department of Agricultural Food and Nutritional Science, University of Alberta, Edmonton, AB T6G 2P5 Canada

## Abstract

**Background:**

In genetic terms, the history of a population can be described by its effective population size over time. We know that domestic cattle have gone through dramatic changes in recent history, including intensifying selection in modern breeding programs and replacement of traditional breeds. In this work, we use linkage disequilibrium-based methods to estimate recent population history from genotype data in Swedish cattle breeds, as well as international Holstein and Jersey cattle data for comparison. We used population genetic simulation to check the inferences.

**Results:**

Our results suggest that the breeds have been effectively large up until recently, when they declined around the onset of systematic breeding. The inferred trajectories were qualitatively similar, with a large historical population and one decline. When comparing simulations from the estimated population histories to real data, the proportion of low-frequency variants in real data was different from what was implied by the estimated population histories, and there was somewhat higher genomic inbreeding in real data than implied by the estimated histories.

**Conclusions:**

The estimates of recent population history of cattle imply that much of the variation we see today is transient, and it will be lost as the populations settle into a new equilibrium, even if efforts to maintain effective population size at current levels are successful.

**Supplementary Information:**

The online version contains supplementary material available at 10.1186/s12711-026-01050-z.

## Background

Population history affects the amount and structure of genetic variation, and thus the efficacy of selection, the accuracy of genomic prediction, and the maintenance of genetic diversity for the future. In population genetic terms, the history of a population can be described as a trajectory of effective population size over time. The effective population size is the size of an idealised population with a rate of genetic diversity loss or increase in inbreeding equivalent to the real population (reviewed by [1]).

Various methods exist for estimating population history from molecular or pedigree data. In this work, we use the linkage disequilibrium-based method GONE [2] to estimate recent population history from molecular data in Swedish cattle breeds, as well as publicly available international Holstein and Jersey cattle data for comparison. A rationale for concentrating on recent population history is that cattle breeds, as we know them today, were created relatively recently. Linkage disequilibrium-based methods perform well for recent history up to 100—200 generations ago [2, 3] and are relatively insensitive to selection [4], but can be biased by population structure, admixture and gene flow [5].

The main commercially important cattle breeds in Sweden are Holstein, Swedish Red and Jersey cattle. Holstein, Red and Jersey cattle are international breeds served by many breeding companies and national and international evaluations. For example, for Sweden, Denmark and Finland, there is a joint Nordic evaluation for these breeds. There are nine Swedish local breeds (listed in FAO-DADIS [6]) with the small ones being subject to conservation efforts by breed societies, supported by the state. The local breeds can be divided into a northern group, including Fjäll cattle (Swedish Mountain cattle), Fjällnära and Bohus Polled cattle, and a southern group including Swedish Red Polled and minor breeds Ringamåla and Väne [7, 8]. Swedish Polled cattle (*Svensk kullig boskap*, SKB, in Swedish) is a combined breed that for historical reasons includes both Fjäll and Swedish Red Polled cattle and has a history of crossing with other breeds. The original Swedish Lowland (*Svensk låglandsboskap*, not included in this study), which diverged before the extensive use of imported Holstein–Friesian genetics, and Swedish Red are also considered among the local breeds, even though Swedish Red is also a commercial breed with gene flow from Danish Red in Denmark and Finnish Ayrshire in Finland.

All local breeds have gradually decreased in census size as they have been replaced by international dairy and beef breeds [9–11], and according to the DAD-IS report, all but Swedish Red are considered at risk. In contrast, commercial breeds frequently have small contemporary effective population sizes [12–14] because they have experienced stronger selection intensity. Some of the local breeds have a known history of admixture. Swedish Red cattle was formed in the late nineteenth century with contributions from Shorthorn, Ayrshire and local Swedish cattle [15, 16]. Swedish Polled (SKB) originated from the merger of breed societies for Fjäll cattle and Swedish Red Polled in 1938 and then from the 1960s also experienced some introgression from other breeds to improve milk production [15]; the samples included in this study are known to have a majority of Fjäll ancestry. The Fjällnära cattle originates from four genetically different herds and in our genomic data we have representatives from all four ancestries [7, 17].

Therefore, the aim of this paper is to estimate the recent population history of Swedish cattle breeds (Bohus Polled, Fjäll, Fjällnära, Red Polled, Ringamåla, Väne, Swedish Polled, Swedish-Holstein Friesian, Swedish Red), with international Holstein and Jersey cattle as comparison. We also used population genetic simulations to check the quality of the inferences that we have made and to simulate consequences for future genetic variation.

## Methods

### Data

For the Swedish cattle breeds, we used high-density SNP chip data generated by [7]. These animals were genotyped on a 150 k Illumina array. For comparison, we extracted samples from Holstein and Jersey cattle from the public release of the 1000 Bull genomes project [18], run 9, downloaded from the European Nucleotide Archive (project accession PRJEB56689). These data, based on Illumina sequencing from many partners, are provided as genotypes in variant call format. We used the filter information provided to include only single nucleotide variants that passed variant quality score recalibration. We used Plink version 1.90b6.21 [19], bcftools version 1.9 [20] and GATK version 4.2.6.1 [21] for filtering and format conversion of genotype data. We included only biallelic single nucleotide variants located on autosomes and that were not monomorphic within breed. The SNP chip positions were lifted to the ARS-UCD1.2 reference genome using UCSC LiftOver, excluding markers that were not unambiguously placed in the new genome. Following the recommendations of the authors of GONE, we did not perform further allele frequency or linkage disequilibrium filtering, as that might bias the estimates. For the 1000 Bull genomes data, we also performed a principal component analysis (Additional File 1 Figure S1) to detect population structure. Two Holstein animals were excluded from the 1000 Bulls genomes because they were extreme outliers in the principal component analysis. Table 1 shows the number of animals and data type for each breed used for estimating population history, the sex distribution and the number of variants retained after filtering. In the case of the 1000 Bull genomes data, we inferred sex using the –impute-sex function in Plink, which is based on inbreeding coefficient of the X chromosome, using a threshold of < 0.2 for female samples and > 0.4 for male. We also used sequence data from 7 Fjäll cattle and 9 Swedish Red Polled cattle, previously published by [8]. These data were not used for population history estimation, but only for comparisons of minor allele frequency spectra and inbreeding coefficients between simulated and empirical data.

**Table 1:** Sample sizes and type of data available for each breed

Breed	Description	Data type	Sample size (females)	Number of variants
Fjäll	Native breed at risk	SNP chip	23 (20)	111,354
Swedish Holstein–Friesian	Commercial breed, Swedish sample	SNP chip	24 (5)	115,127
Swedish Red	Native breed, not at risk, commercial	SNP chip	25 (5)	115,016
Holstein 1000 Bulls	Commercial breed, international sample	Sequence	144 (58*)	13,371,827
Jersey 1000 Bulls	Commercial breed, international sample	Sequence	77 (5)	11,056,218
Red Polled	Native breed at risk	SNP chip	18 (8)	112,029
Fjällnära	Native breed at risk	SNP chip	16 (14)	109,327
Ringamåla	Native breed at risk	SNP chip	13 (11)	105,447
Swedish Polled (SKB)	Native breed at risk	SNP chip	12 (0)	109,644
Väne	Native breed at risk	SNP chip	9 (4)	94,070
Bohus Polled	Native breed at risk	SNP chip	7 (4)	106,755

^*^There were also 5 animals in the Holstein 1000 Bull genomes data that could not unambiguously be assigned a sex based on inbreeding coefficient of the X chromosome

### Population history inference with GONE

To infer recent population history over the last 200 generations, we used the GONE software version 29/08/2021 [2]. GONE uses an equation to predict pairwise linkage disequilibrium for a given population history, and a genetic algorithm to fit a population history to the data. We applied GONE to SNP chip or sequence data from each population without allele frequency filtering. In cases where there are more than 50,000 variants on a chromosome, GONE randomly subsets the variants. Following recommendations of the authors, we limited the estimated population history to the most recent 200 generations. We also summarised the population histories for each breed by finding the generation with the biggest change in effective population size from the previous generation, and the mean effective population size in generations before this decline.

To create uncertainty intervals for the estimates from GONE, we applied the method to random subsets of the data. We tested two procedures, either subsampling by chromosomes by leaving one chromosome out each time or subsampling by sample by leaving one sample out each time. We then constructed an interval by taking the maximum and minimum estimate from those subsampled replicate analyses. We applied both methods to simulated data (Additional File 2 Figure S2) where they performed similarly and we used the leave one sample out method on the real data. For the SNP chip data, we ran as many replicates as there were samples, leaving one out each time. For the sequence data, we used 40 random replicates each leaving out randomly selected samples to reduce the computation time.

In addition to estimates by GONE, we also tested the classical equation-based model for estimating population history from linkage disequilibrium [22–24], as implemented in SNeP version 1.11 [25]. However, simulations showed that SNeP performed poorly under circumstances where the effective population size has recently declined rapidly (Additional File 3 Figure S3), like we expect in cattle breeds, and it was not used for the main analysis.

### Population genetic simulation

To check the validity of the population history inferences, we performed population genetic simulations. We performed one set of simulations to check the performance of GONE on data from simple population histories similar to what we expect in cattle, i.e., one or several recent declines and the full Holstein population history estimated by [26]. We also simulated data from the population histories estimated by GONE for a subset of the breeds (Fjäll, Red Polled, Swedish Holstein–Friesian, Swedish Red, Holstein, Jersey) in this paper and compared them to real data with respect to statistics not directly used for inference (as recommended by [27]). Simulations were performed with msprime version 1.3.1 [28].

In order to avoid problems with coalescent simulation of small effective population sizes, which tend to underestimate long-range linkage disequilibrium in particular [29], we used a hybrid simulation strategy with coalescent simulation in the large historical population followed by discrete Wright—Fisher simulation for the last 200 generations, adjusting the population size at pre-defined times. Finally, we extracted a population sample from the last generation and extracted genotypes at either a simulated 100 k SNP chip or used all variants to simulate whole-genome sequence data.

### Simulations to check the performance of inference methods

To check the performance of GONE on population histories similar to what we expect in cattle, we applied GONE to data simulated from these four population histories:

- The cattle population history estimated by MacLeod et al. [26].
- Decline: A simple declining population history with a large historical population size of 5000 followed by a decline 50 generations ago to 100.
- Two declines: A declining population history with two steps, with a large historical population size of 5000 followed by a decline 100 generations ago to 2500 and another decline 50 generations ago to 100.
- Recovery: Decline and recovery, with a large historical population size of 5000 followed by a decline 50 generations ago to 50, and an increase 20 generations ago to 100.

In each simulation replicate, we sampled 20 individuals from the last generation and 100 k SNPs to represent a SNP chip and ran population history inference. We replicated each simulation 10 times and plotted the estimated and true simulated population history together. We also calculated the mean absolute deviation of the estimated population histories compared to the true simulated values for each generation. This gives insight into the error profile of the methods when applied to declining population histories, such as those anticipated in cattle populations.

### Simulation checks of estimated population histories

To investigate the fit of the estimated population histories to real data, we simulated data from the population histories estimated for from Fjäll, Red Polled, Swedish Holstein–Friesian, Swedish Red, Holstein, Jersey and the published Holstein population history of MacLeod et al. [26]. For the population histories estimated with GONE, we used the effective population size 200 generations ago as a stable historical population size, which was simulated using coalescent simulation. For the last 200 generations, we adjusted the effective population size each generation to the value estimated by GONE and used discrete Wright—Fisher simulation.

From these simulated data, we estimated population genetic statistics and compared them to statistics from real data for the same breeds. For this comparison, we used the SNP chip data from Swedish breeds and the 1000 Bull genomes data for Holstein and Jersey, i.e., the same data that was used for inference. We also included sequence data from 7 Fjäll cattle and 9 Swedish Red Polled cattle, previously published by [8], which were not used for population history inference since we considered the sample sizes inadequate.

We extracted simulated genotype data from the same number of simulated animals as in the empirical data. For comparison with SNP chip data, we subsampled the simulated SNPs randomly to a 100 k SNP chip. For comparison with sequence data, we used all simulated variants.

From both empirical and simulated data, we used Plink to estimate:

- Minor allele frequency spectrum calculated in frequency windows. We used windows of 0.1 for most breeds and of 0.05 for the 1000 Bull genomes Holstein and Jersey cattle, where the sample size is greater.
- Inbreeding coefficient based on homozygous genotypes ${F}_{HOM}$, which is a method of moments estimate of inbreeding based on comparing the observed count of homozygous genotypes in an animal compared to the expectation from Hardy—Weinberg proportions.
- Inbreeding coefficient based on runs of homozygosity ${F}_{ROH}$. Runs of homozygosity were detected with Plink. We used the default settings of requiring that a run contain at least 100 SNPs, at least one SNP per 50 kbp, no more than a 1000 kbp gap between SNPs, and have a total length of at least 1 Mbp. For SNP chip data, we used the default settings for scanning windows, namely at least 50 SNPs per window, at most 1 heterozygous and 5 missing calls per windows, and a homozygous window threshold of 0.05. For sequence data, we used more permissive settings for scanning windows, allowing for 3 heterozygous and 10 missing markers per window for sequence data. The inbreeding coefficient was calculated by dividing the total length of runs in an animal by the autosomal genome length.

We plotted simulation replicates against the estimates from real data. This gives us insight into how well the estimated population histories match real data, based on features not directly used in population history inference.

### Simulation of future genetic variation

To explore the consequences of the population histories for genetic variation, we ran simulations where each population history was extended with another 200 generations at the estimated final effective population size for a total of 400 generations. For this simulation, we only included one chromosome, the length of cattle chromosome 1. Every 10 generations, we sampled 20 individuals and estimated the pairwise nucleotide diversity ($\pi$) within the sample, which we plotted to illustrate the decline in genetic diversity over time. The diversity calculation was performed with tskit version 0.5.6 [30].

## Results

### Estimated population histories

**Fig. 1:**
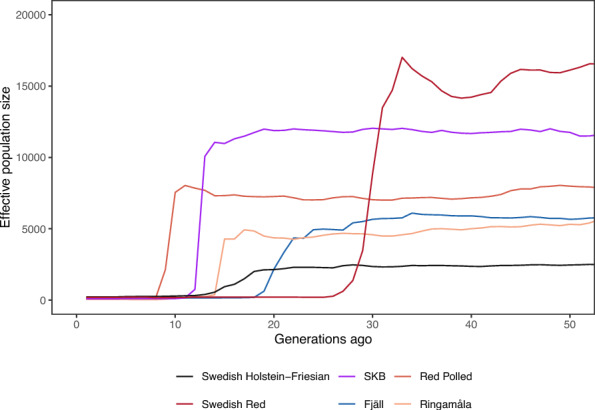
Population histories of Swedish cattle breeds, estimated by GONE from SNP chip data. The horizontal axis shows time in generations ago, running backwards. The vertical axis shows the estimated effective population size. The figure shows breeds with a sample size greater than 10, excluding the Fjällnära breed

**Fig. 2:**
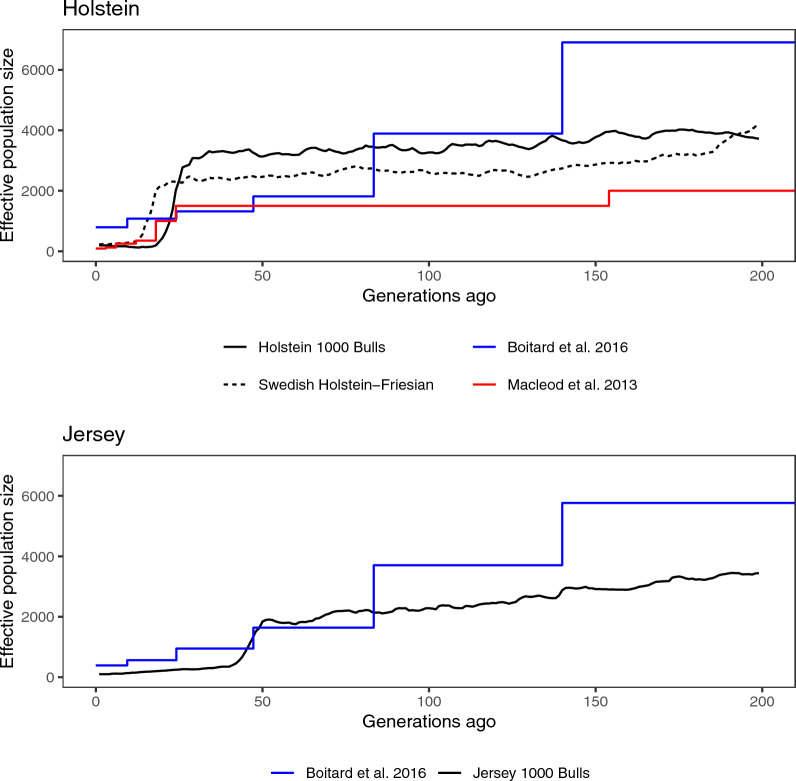
Population histories of international Holstein and Jersey cattle, estimated by GONE from 1000 Bull genomes genotypes. The dashed line shows Swedish Holstein—Friesian for comparison. The horizontal axis shows time in generations ago, running backwards. The vertical axis shows the estimated effective population size. The red and blue lines show published population histories

The estimated population histories all showed a qualitatively similar pattern, with historical population size in the thousands, and a recent decline. Figure 1 shows the estimates for the Swedish breeds based on SNP chip data, showing the breeds with at least 10 animals sampled except for Fjällnära cattle, which showed a likely artefactual history indicative of poor model fit. We show only the last 50 generations, because there was little change in the estimated effective population size before that, and this likely reflects loss of power to resolve earlier population history rather than being evidence of constant historical population size. Figure 2 shows the estimates for Holstein and Jersey samples from the 1000 Bull genomes data, with comparison to previously published histories [26, 31]. The horizontal scale in these figures go further back in time to facilitate comparison with published population histories. Additional Data 4 Figure S4 shows the estimated history for every breed on its own scale with uncertainty intervals generated by random subsampling, leaving one sample out each replicate. These uncertainty intervals suggest that the variation in estimated historical population size shown in Fig. 1 is largely due to uncertainty in estimation rather than being good evidence that the historical population sizes are different. Additional file 5 Table S1 shows the approximate timing of the decline (ranging from 9 generations for Red Polled to 47 generations for Jersey), the mean effective population size before the decline (ranging from 2,608 for Jersey to 20,021 for Swedish Red). All estimated histories are provided in Additional File 6 Table S2.

**Fig. 3:**
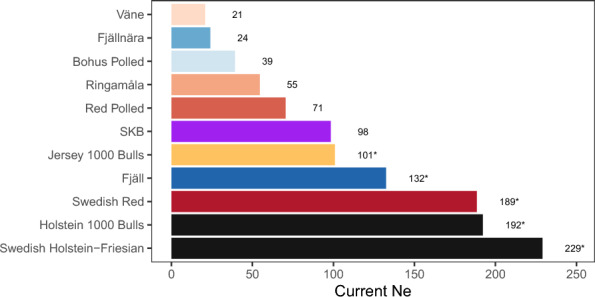
Estimates of current effective population size from GONE. The bars show the estimated current effective population size (i.e., from the most recent generation) estimated by GONE. The estimates marked with an asterisk correspond to breeds with a sample size above 20

The estimated current effective population sizes of all breeds ranged from around 20 to around 200 (Fig. 3). The numerically small traditional breeds, such as Väne at an effective population size of 20, Fjällnära at 24 and Bohus Polled at 39, tended to be smaller than the commercial breeds, with Swedish Red at 189, and Swedish Holstein–Friesian at 229. However, Fjäll cattle was estimated to have higher effective population size than Jersey, with 132 compared to 101.

### Performance of GONE on simulated data

**Fig. 4:**
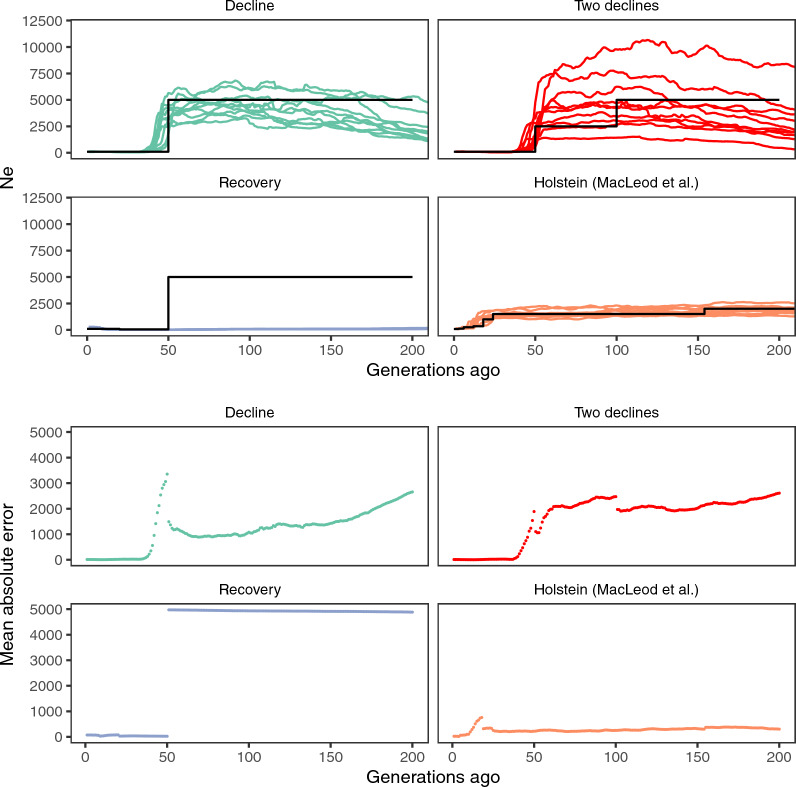
Population history inference with GONE applied to simulated data. The black lines show the true values of the simulated population histories. The coloured points show estimated population histories from 10 simulation replicates each. The upper panels show estimated effective population size and the lower panels show the mean absolute deviation between real and estimated effective population size in each generation. The simulated scenarios are: simple population histories with one (“Decline”) or two declines (“Two declines”), a simple population history with a decline followed by a recent recovery (“Recovery”) and a published population history for Holstein cattle (“Holstein MacLeod et al.”)

Inference on data simulated from known population histories suggested that the method was able to infer the shape of population histories with recent declines, but that there was substantial uncertainty in historical population size. Figure 4 shows estimated and simulated population histories together. The mean absolute deviation from the true simulated effective population size was around 1000 from 50 to 100 generations ago with one decline, larger with two declines, but smaller in the MacLeod et al. history. There was a systematic bias in estimates from the simple decline histories, where in earlier generations (particularly pronounced before generation 150), the effective population size appeared to be spuriously growing. In the case of a recent extreme bottleneck with recovery, the method was unable to detect the previous population decline before the bottleneck, and instead estimated a spuriously small historical effective population size.

### Fit of estimated population histories to real data

**Fig. 5:**
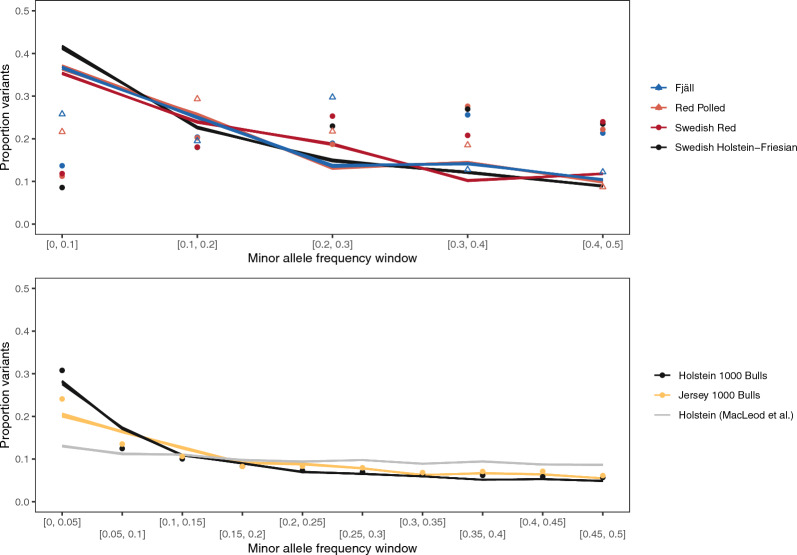
Comparison between the allele frequency distribution in simulations from estimated population histories and real data. The lines show replicated simulations from the estimated population histories. Colours correspond to breeds. The round points show population genetic estimates from real data. In the case of the top panel, this is SNP chip data, and in the bottom panel variants from 1000 Bull genomes sequence data. In the top panel, the open triangles show estimates from whole-genome sequence data from Fjäll and Red Polled cattle

Simulations from estimated histories showed discrepancies between the allele frequency distribution implied by the estimated and the allele frequency distribution observed in real data. Figure 5 shows minor allele frequency distributions for data simulated from estimated population histories and from real genotype data. In the SNP chip data from the Swedish cattle breeds, the simulated data showed an overrepresentation of rare variants compared to the real data. In sequence data from Fjäll and Red Polled cattle, there was still an overrepresentation, but it was smaller. In the sequence data from the 1000 Bulls, where sample sizes were bigger, there was an underrepresentation of rare variants in the simulated data compared to real sequence data. Simulations from the population history of MacLeod et al. [26] also showed an underrepresentation of rare variants compared to real data.

**Fig. 6:**
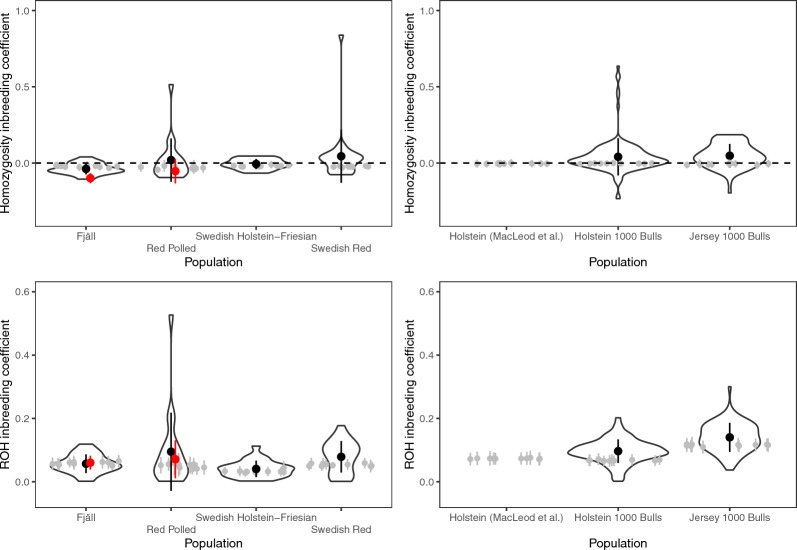
Comparison between the genomic inbreeding in simulations from estimated population histories and real data. The violin plots show estimates from real data, with black points and bars showing the mean and standard deviations of the genomic inbreeding coefficients from real data. The grey points and error bars show means and standard deviations of the genomic inbreeding estimated from simulated data. For the Swedish cattle, where inference is based on SNP chip data, the red points and bars show estimates from whole-genome sequence data from Fjäll and Red Polled cattle

The level of inbreeding in simulated data was somewhat lower than the level observed in real data. Figure 6 shows genomic inbreeding coefficients estimated from simulated data compared to real genotype data. Except for Fjäll cattle, the inbreeding coefficients estimated from data simulated based on the inferred population histories are, on average, lower than those observed in real data. The same was true for the population history of MacLeod et al. [26] compared to Holstein data from the 1000 Bull genomes project. In particular, there were real animals with very high inbreeding coefficients, that were not generated by simulated data. In most cases, the mean of the simulated inbreeding coefficient was within a standard deviation of the real value.

### Consequences for future genetic variation

**Fig. 7:**
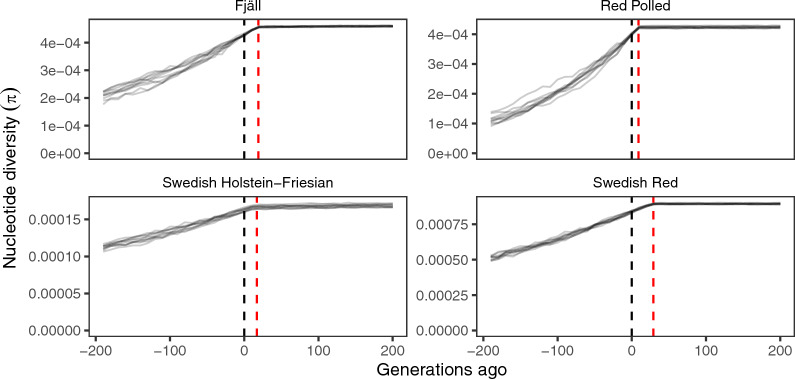
Recent and future genetic diversity trajectories of Swedish cattle breeds, based on simulations of their estimated population histories. The panels show simulated nucleotide diversity for Fjäll, Red Polled, Swedish Holstein–Friesian and Swedish Red. The lines show estimates of nucleotide diversity from samples of 20 individuals taken every 10 generations. For consistency with the other graphs, time is shown running backwards along the horizontal axis, with zero as the current time, indicated by the black dashed line. Thus, the negative generation numbers represent future generations. The red dashed line represents the estimated time of maximum decline for each breed

The estimated population histories imply populations that are not in equilibrium, and where there is transient genetic variation that will be lost over a long time. Figure 7 shows simulated trajectories of nucleotide diversity from four of the estimated population histories, extended by 200 generations where the population remains at the estimated current effective population size. The simulations showed nucleotide diversity remaining relatively constant up until the population size decline, and a steady decrease in diversity in the future, where the populations would not reach equilibrium within 200 generations.

## Discussion

In this work, we estimated the recent population history of Swedish cattle breeds, with international Holstein and Jersey cattle as comparison. Our results suggest that these breeds have been effectively large up until recently, when they declined around the onset of systematic breeding. Secondarily, we used population genetic simulation to check the quality of our inferences. Comparisons between simulations derived from estimated population histories and empirical data revealed some discrepancies. There was a higher proportion of low-frequency variants in 1000 Bull genomes data than is implied by the estimated population histories, and somewhat higher genomic inbreeding in real data than implied by the estimated histories.

We will discuss potential biases that affect these results, correspondence to known events, the relevance to quantitative genetic simulation of cattle breeding, and the implications for cattle genetic resources.

### Sources of error and bias

There are several sources of uncertainty and bias in effective population size estimation that should be kept in mind when interpreting our results.

The largest estimated historical effective population sizes in our analysis come from breeds that have a history of admixture and population structure. This is a known bias in the method [5], which assumes a sample from a closed and well-mixed population. Thus, the implausibly high historical effective population sizes of Swedish Red Cattle (around 20,000), Swedish Polled (SKB) (around 13,000) and Red Polled (around 10,000) should likely not be taken at face value. Historically, Swedish Red cattle was formed by crossing imported Shorthorn and Ayrshire with local Swedish cattle in the eighteenth century [15, 16]. This scenario is similar to the creation of a synthetic breed simulated by Novo et al. to evaluate GONE’s performance. Their results suggest that the estimated timing of admixture, i.e., the inferred decline, is accurate but that the historical effective population size is overestimated compared to the combined size of the founding population [5]. More recently, the Swedish Red breed shares a genetic evaluation and semen providers with Danish and Finnish Red. Swedish Polled, on the other hand, is a product of administrative concerns, as the two breed societies for Red Polled (SKB) and Fjäll were joined in 1938 [15]. Admixture between Fjäll-derived and Red Polled-derived SKB cattle were probably not such a big factor, as the breeds are quite distinct both in terms of ancestry and phenotype. However, there was conscious crossing with other breeds, including Swedish Holstein—Friesian, Jersey and Finnish cattle [32]. Thus, both population structure and admixture likely contribute to inflating the estimated historical effective population size. The Red Polled breed has historical population structure, with multiple ancestries including important influence from Norwegian and Finnish Red Polled cattle [15].

Fjällnära cattle showed a likely artefactual estimated population history, with an exceptionally large recent population history and a steep decline. This pattern is similar to what the creators of GONE have shown to happen when there is strong population structure within the sample, which breaks the assumptions of the method [5]. Indeed, the Fjällnära breed is known to have strong population structure, with four distinct subpopulations [17].

Both uncertainty intervals constructed by resampling and results from running the GONE simulated data suggests that there is substantial uncertainty in the magnitude of historical effective population size. In particular, the effective population size estimates from most of the local breeds are based on fewer than 20 individuals and should be regarded as indicative only. The uncertainty intervals for historical population size covers several thousand for most breeds and are extremely wide for the breeds with the smallest sample sizes. We should also emphasise that these are not proper confidence intervals with guaranteed coverage, meaning that the uncertainty is likely even greater than they suggest. In this paper, we tested two ways of generating resampling intervals, either by leaving out samples or by leaving out chromosomes, with comparable results. There are possible refinements that could be explored further, such as blockwise resampling where parts of the genome are held out rather than whole chromosomes.

In addition to uncertainty about the size of the historical population, our simulations clearly show that this method struggles to reconstruct earlier population history before the decline. This is intuitive, as drift in the extremely small effective population size after a decline is likely to obscure traces of earlier population genetic processes. Simulations with multiple declines (the simple “Two declines” population history and the Holstein history of [26]) and with decline followed by recovery, showed how estimated histories effectively only captured the last population history change.

Furthermore, the uncertainty depends on the features of the true historical populations size. In simulations, where the true historical population size was known, the uncertainty was more pronounced in the simple decline scenario which has a higher historical population size, and even more in the simulated scenario with two declines within a relatively short time frame, compared to simulations of the MacLeod et al. [26] population history. Fully exploring this error and its scaling with population size would require more extensive simulations with ranges of parameters. Suffice to say that one should treat the estimated historical population sizes with caution. In simulations of declining populations, we also saw a bias in the early part of the population history where the effective population size appears to be growing when it was in fact simulated to be constant. This suggests that similar patterns for example in the estimated population history of Ringamåla cattle may not be trustworthy. In general, the error is higher for earlier generations, which should be expected.

The simulated scenario of a recent bottleneck with recovery suggests that the method may struggle with inferring the population history after an extreme bottleneck. In the bottleneck scenario we simulated, the estimated population history detected a recent increase from a long-time low historical effective population size, suggesting that such an extreme bottleneck effectively erases information about previous historical population size. In the estimated histories, only Bohus Polled cattle, which is indeed a very small breed that has likely been subject to an extreme recent bottleneck, showed a pattern similar to this. However, the Bohus Polled history is also based on a small sample size and shows other likely artefactual features like a recent instantaneous increase in effective population size, which could potentially be related to the use of Fjäll bulls in efforts to conserve the Bohus Polled breed in the 1990s. As a corollary, these results suggest that the bottlenecks in the other Swedish breeds have not been as extreme as the simulated case. However, we only simulated one parameter combination, and therefore caution is warranted.

Furthermore, all the Swedish cattle samples used in this study are non-random samples of their respective breed. Therefore, we should expect, in general, an upward bias in historical effective population size. Similarly, the 1000 Bull genomes data combine samples from many different partners all over the world. Such international breeds can be regarded as metapopulations consisting of connected subpopulations, and as such, would be expected to show a higher metapopulation effective population size than the effective size of the constituent subpopulations [33, 34]. For example, structure within the Holstein breed has been identified within and between countries [35, 36]. This may explain why the estimated current effective population size, around 200, appears high compared to Holstein estimates from breeding programs [12, 14]. For subdivided populations, the authors of GONE suggest [5] that one may split the data, and estimate the population history for subpopulations separately. However, when inspecting principal component plots for Holstein and Jersey data from 1000 Bull genomes, we find no clear clustering that could be used to divide the data in such a way. Furthermore, the composition of sexes in the samples is not even and varies between breeds, which might bias the estimates since the male effective population size is expected to be smaller than the female. For example, the 1000 Bull genomes samples of Jersey cattle was highly biased towards males, whereas some of the local Swedish breeds had female-biased samples.

Simulation-based checks of estimated population histories show discrepancies between the real allele frequency distributions and those implied by simulations. For the Swedish cattle data, there was an overrepresentation of low-frequency variants implied by the estimated history. However, in the case of SNP chip data, this discrepancy may be due to ascertainment bias, where SNP chips are designed to be biased towards common variants. Further, the sequence data from Fjäll and Red Polled cattle come from small enough sample sizes (seven and nine cattle respectively) that they cannot estimate low frequencies well. In the case of the 1000 Bull genomes data, where sample sizes are greater, the estimated histories imply a smaller proportion of low-frequency variants than observed in real data.

In addition to GONE, there are earlier methods for population history inference from linkage disequilibrium that are occasionally still used in the animal genetics literature. In the early phase of this work, we attempted to use one of these classical methods, implemented in the software SNeP [25], as an alternative to GONE (Additional file 3 Figure S3). While the recent effective population size estimates are of a similar magnitude to GONE, and rank breeds roughly in a similar order, our simulations suggest that the classical method implemented in SNeP do not perform well for population histories with recent declines and instead infer gradual declines and underestimates historic effective population size. Problems with this method were pointed out already by Corbin et al. [24] and repeated by the authors of SNeP [25]. These methods are not suited to populations that had rapid changes in effective population size recently, such as most domestic animals. In the case of Swedish cattle breeds, we know that recent declines have happened based on history, independent of molecular estimates.

### Correspondence to known events and previous estimates

The approximate time of the estimated declines occurred around ten to twenty generations ago, which corresponds to the mid to early twentieth century, assuming a generation time of around five years and that the cattle sampled were born in the late 1990s to early 2000s. This roughly corresponds to the onset of systematic breeding or the later decline due to the replacement of traditional breeds by international breeds. For example, Fjäll cattle the inferred decline occurs 19 generations or approximately 100 years ago (assuming a generation time of five years), and a breed standard was established in 1893 [15]. For Red Polled, the inferred decline occurs 9 generations or approximately 50 years ago; in the mid twentieth century the breed declined down to almost going extinct in the 1970s. For Swedish Red Cattle, the inferred decline occurred 29 generations or approximately 150 years ago. The original two breed societies for what would become Swedish Red were formed in 1892 and 1898. They were both already working with animals that were mixed between Shorthorn, Ayrshire and local Swedish cattle [16], suggesting that admixture had happened earlier. One exception is Jersey cattle, where the inferred decline occurred 47 generations or approximately 200 years ago. This is consistent with the breed’s island origin, where the population has been closed with no importation allowed from 1789 and a breed standard was established in 1834 [37].

In the case of Holstein and Jersey cattle, we can compare our estimated population histories to published histories [26, 31]. In both cases, the previously published histories go further back in time than the 200 generations used for our estimates, going back to 33,154 and 130,000 generations ago, respectively. Compared to previous histories, both our Swedish and international Holstein estimates suggested a higher effective population size more recently. The decline was more dramatic compared to MacLeod et al.’s [26] estimates, but less dramatic than Boitard et al.’s estimates [31], where the latter results in an unexpectedly high current effective population size close to 1000. Further back in the time, the discrepancy between our estimates and previously published estimates grows bigger, which is to be expected as GONE is more accurate for recent than earlier history.

As mentioned above, our estimated current effective population sizes for Holstein appear high compared to current estimates from breeding programs (192 and 229 vs less than 50 in several breeding programs [12, 14]). Our estimate for Swedish Red cattle is lower (189 vs 226) compared to the pedigree-based analysis of Nyman et al. [13]. The estimate for Swedish Polled (SKB) was also lower than their pedigree-based estimate (98 vs 166). The pedigree-based estimates pertain to the whole pedigree based on data from 1960 to 2018, and in our estimated population histories we infer little change during this period. Thus, the estimates should have a comparable time frame. For most of the local Swedish breeds, pedigree-based estimates of effective population size are lacking. Estimating effective population size with standardised pedigree-based methods would be a valuable contribution to monitoring of these breeds. Compared to estimates of recent effective population size of Finnish Ayrshire, a breed contiguous with Swedish Red, our estimate falls in between their estimates based on pedigree and genomics (165 and 266) [38].

The historical generation time of cattle is uncertain, as it changes with breeding practices. When discussing the timing of events above, we used a generation interval of 5 years, which is consistent with estimates from Red, Holstein and Jersey cattle before genomic prediction [13, 14]. However, there are several reasons why generation time may have changed with time. Historically, before the onset of systematic breeding, generation time on the male side may not have been that long, due to the cost of keeping bulls. However, with the classical breeding structure of progeny testing to evaluate bulls, proven bulls lived long, and generation times especially on the male side were long. After the introduction of genomic selection around 2010, the generation times of commercial cattle breeds have dramatically declined [14, 39]. The Swedish samples used in this study are not affected by this change, as the Swedish Red and Holstein—Friesian cattle were born before genomics, and the small breeds do not have genomic breeding. However, when viewing the 1000 Bull genomes estimates, one should keep in mind that the most recent generations likely have shorter generation intervals. This also affects the predictions of future decline in genetic variation. Commercial breeds like Holstein cattle have shorter generation times than a local breed under conservation breeding, with correspondingly higher rate of loss of variation over time.

Both Fjäll cattle and Red Polled cattle have suffered documented genetic crises in the latter half of the twentieth century. The breeds declined severely, and efforts to rescue them were undertaken starting in the 1980s and 1990s [15, 32]. These efforts include collection and use of cryopreserved semen from old bulls as well as attention to ancestry and diversity rather than selection for trait improvement (as expressed in breeding plans [40–43]). The estimates of recent effective population sizes, which are comparable to estimates for international breeds, confirm that these efforts have to some extent been successful. For example, for Fjäll cattle, we estimated a current effective population size of 132 that appeared not to be declining further. We would like to emphasise that an inferred stable effective population sizes after the decline does not mean that there is no concern. These breeds are considered at risk, have decreasing census sizes, and serious concerns with economic viability for farmers.

At the same time, we note that our estimated population histories for these breeds do not show multiple declines, bottlenecks and recoveries. Instead, the estimated population histories simply appear like simple declines from a historically stable effective population size. This suggests that the method is unable to reconstruct complicated changes in population history at sufficient resolution. As discussed above, our tests with simulated data confirm this, showing how GONE could not disentangle multiple declines in effective population size. In the breeds that suffered crises, it may be that the rescue efforts themselves may contribute to obscuring the traces of this history in the molecular data. In the case of deeper history before the decline, the decline itself may obscure earlier patterns of effective population size change.

Also in Swedish Holstein and Red Cattle, genotyping of old bulls has shown that there has been fluctuations in inbreeding rate over the latter half of the twentieth century, where inbreeding decreased from the 1960s to 1980s and later increased again in Holstein but remained relatively stable in Red cattle [44]. That this signal can be recovered in historical genotypes suggests that it would be possible to estimate a more fine-grained population history from time series data.

### The relevance to quantitative genetic simulation

Our simulation-based checks of our population history estimates suggest a higher proportion of rare genetic variants than implied by previous estimates [26], and that our estimated histories from 1000 Bull genomes data still lead to an underrepresentation of rare variants compared to the real genotypes. This has implications for the genetic architecture of complex traits in simulation, suggesting that simulated allele frequency distributions are often too uniform.

Such differences may have little impact on simulations that aim at predicting the short-term response to selection in different breeding schemes. To a first approximation, response to selection depends on genetic variance, not details of genetic architecture. However, such properties may matter when simulations aim to describe mechanisms of responses to selection, genomic prediction accuracy and evolution of genetic variance, which may depend on linkage disequilibrium and genetic architecture [45–48]. As an example, MacLeod et al. [49] found that when simulating genomic prediction with whole-genome sequence data, simulating a declining population history rather than a constantly large population destroyed the benefit of whole-genome sequence data.

With recent developments in population genetic simulation, using a more realistic population history when relevant is not particularly difficult. Tools like the community-maintained library of population histories stdpopsim [50, 51] will likely make it easier in the future. However, when simulating more realistic population histories, especially when attempting to simulate whole genomes or in combination with complications such as selection, computational constraints become a real problem. Our simulations comparing estimated population histories to real data suggest that is possible to create simulated data with similar or better agreement with reality—at least with respect to the allele frequency distribution and genomic inbreeding—than previous estimates without simulating ancient population history. This may save some time and computational resources.

### Implications for genetic resources

Due to a lack of resolution for recent events like multiple declines and bottlenecks, combined with substantial uncertainty, this type of molecular population history inference appears to have limited use in applied conservation of local breeds. For tracking generation-to-generation effective population size, one would need other methods that could be based on pedigree registration or on molecular monitoring.

It should be uncontroversial that genetic diversity of farm animals is decreasing. Our results suggest that, for these breeds, a large decrease in effective population size has happened over the last century, after the onset of systematic breeding. Effective population size has dramatically decreased both in numerically small breeds under conservation efforts and in international commercially used cattle breeds, and domestic cattle are now fragmented into effectively small populations. Even if current breeding programs succeed in limiting the future decrease in effective population size and maintain the current size, genetic variation will continue to decline for a long time. Much of the variation we see today is transient, and it will be lost as the populations settle into a new equilibrium. This, however, will take hundreds of generations.

It is an open question to what extent this loss of variation will impede response to selection or viability. Rules of thumb for viable effective population size are often cited as recommendations in conservation genetics [52, 53]. Short-term recommendations of effective population size above 50 or 100 are based on assumptions about tolerable levels of inbreeding depression over the next few generations. The smaller breeds in this study are below these thresholds. Long-term recommendations of effective population size above 500 or 1000 are based on assumed equilibrium between mutational variance and loss of additive genetic variance in an infinitesimal model. On the other hand, Hill argued, using similar quantitative genetic arguments, that the supply of mutation in a population of effective size of 100, may be sufficient for a sustained response to directional selection [54]. This depends on parameters that are hard to know, namely the mutational variance of quantitative traits, not just molecular mutation rates; our simulations, in contrast, only include neutral evolution. The main difference between the approaches is that Hill assumed a higher mutational variance. Current effective population sizes of these cattle breeds are mostly close to or below 100, and definitely below 500. Looking to commercial breeds where there is data on quantitative traits, dairy cattle are not yet facing inbreeding depression that exceeds genetic gain [55]. There are some signs of loss of genetic variance for breeding goal traits in pigs and Simmental cattle [56, 57], but not in a study of Holstein dairy cattle [58] and a chicken breeding program [59].

## Conclusions

Our results suggest dramatic declines in effective population size around the onset of systematic breeding, both in commercially used breeds and threatened native breeds that are under conservation efforts. As expected, the current effective population sizes are close to or below suggested thresholds for long-term viability of populations. The population histories we estimate imply that the levels of genetic variation within cattle breeds will keep declining for hundreds of generations, even if there are no further declines in effective population size.

## Supplementary Information

Additional file 1: Fig. S1. Principal component analyses of 1000 Bull genomes data. Scatterplots of the first and second principal component from Holstein and Jersey datafrom the 1000 Bull genomes dataset.

Additional file 2: Fig. 2. Testing of uncertainty intervals for GONE estimates. The panels show uncertainty intervals for 10 replicates of the simple decline simulation, constructed either by leaving one chromosome out or leaving one sample out. The black line shows the estimate from the full data. The blue line shows the true simulated population history. The bottom panel shows the average coverage of the intervals across generations, i.e., the fraction of replicates where the interval covers the true simulated value in that generation.

Additional file3: Fig. S3. Evaluation of population history inference with SNeP. A) Population history inference with SNeP applied to simulated data. The black linesshow the true values of the simulated population histories. The coloured points show estimatedpopulation histories from 10 simulation replicates each. The simulated scenarios are simplepopulation histories with one (“Decline”) and a published population history for Holstein cattle(“Holstein MacLeod et al.”). B) Population histories of Swedish cattle breeds, estimated by SNePfrom SNP chip data. C) The bars show the estimated current population history (i.e., from the mostrecent generation) of Swedish cattle breeds with SNP chip data estimated by SNeP.

Additional file4: Fig. S4. Population histories estimated by GONE. The horizontal axis shows time in generations, running backwards. The vertical axisshows the estimated effective population size. The shaded areas show uncertainty intervals made bydropping one sample at a time. This figure includes all estimated trajectories on their own scale.

Additional file5: Table S1. Summaries of estimated population histories. Time of the decline, estimated as the generation with the biggest decline in effectivepopulation size from the previous generation, and the mean effective population size before thedecline, from population histories estimated by GONE.

Additional file6: Table S2. Estimated population histories. The comma separated value file contains estimates from GONE for each breed. Thefirst column (“Breed”) contains the name of the breed. The second column (“Generation”) containstime in generations ago. The third column (“Ne”) contains the estimated effective population size.

## Data Availability

The SNP chip data are available in the Dryad repository at 10.5061/dryad.wdbrv15j4. The 1000 Bull genomes data are available from the European Nucleotide archive at project accession PRJEB56689. The sequence data from Swedish cattle are available from the European Nucleotide archive at project accession PRJEB60564. The scripts for data analysis are available at https://www.github.com/mrtnj/cattle_population_history.
