# Correction: Recent population history in Swedish cattle breeds

**DOI:** 10.1186/s12711-026-01059-4

**Published:** 2026-06-03

**Authors:** Dolapo Adepoju, J. Ingemar Ohlsson, Tomas Klingström, Elisenda Rius-Vilarrasa, Anna M. Johansson, Martin Johnsson

**Affiliations:** 1 Department of Animal Biosciences, Swedish University of Agricultural Sciences, Box 7023, 750 07 Uppsala, Sweden; 2 Växa Sverige, 104 25 Stockholm, Sweden; 3 Beijer Laboratory for Animal Science, Swedish University of Agricultural Sciences, Box 7024, 750 07 Uppsala, Sweden; 4 Present Address: Department of Agricultural Food and Nutritional Science, University of Alberta, Edmonton, AB T6G 2P5 Canada

**Correction to: Genet Sel Evol 58, 27 (2026) ** 10.1186/s12711-026-01050-z

Following publication of the original article [1], the authors identified a typesetting error in the author name of J. Ingemar Ohlsson.

The incorrect author name is: JIngemar Ohlsson

The correct author name is: J. Ingemar Ohlsson

The author group has been updated above and the original article [1] has been corrected.

## References

[1] Adepoju D, Ohlsson JI, Klingström T, et al. Recent population history in Swedish cattle breeds. Genet Sel Evol. 2026;58:27. 10.1186/s12711-026-01050-z. 42177417 10.1186/s12711-026-01050-z PMC13202900

