# Supplementary figures and images for: Recent population history in Swedish cattle breeds
Source: Genet Sel Evol. 2026 May 24;58:27. doi: 10.1186/s12711-026-01050-z (PMC13202900; doi:10.1186/s12711-026-01050-z)

Holstein

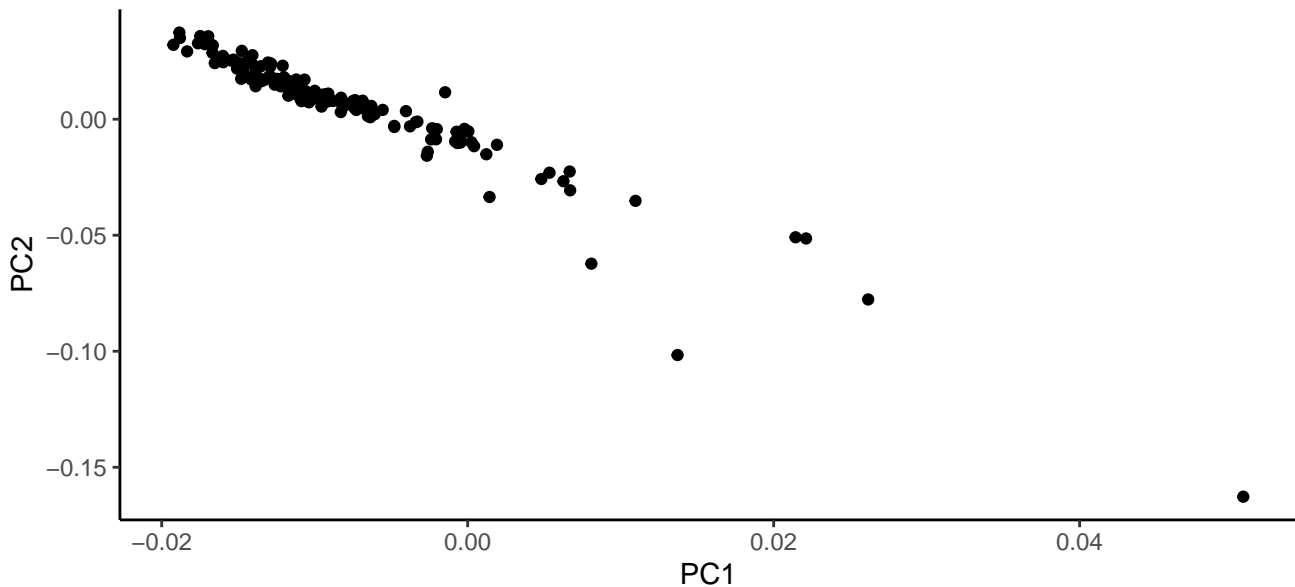

Jersey

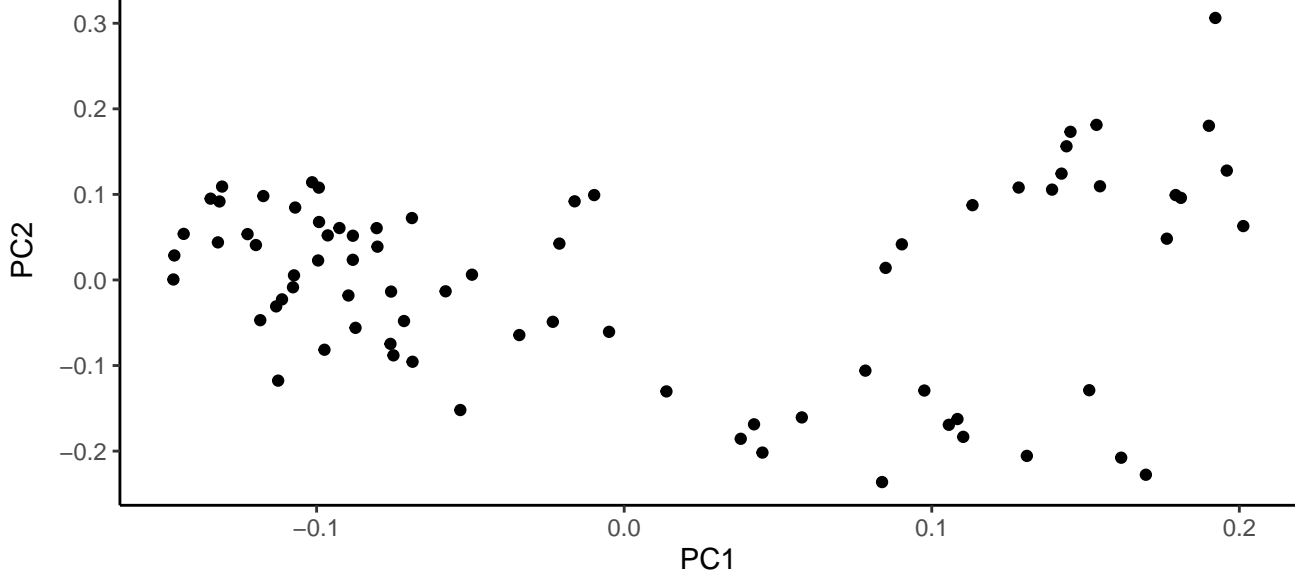

Supplement: Supplementary file 1 — Additional file 1: Fig. S1. Principal component analyses of 1000 Bull genomes data. Scatterplots of the first and second principal component from Holstein and Jersey datafrom the 1000 Bull genomes dataset. [file 12711_2026_1050_MOESM1_ESM.pdf]

Leave one sample out

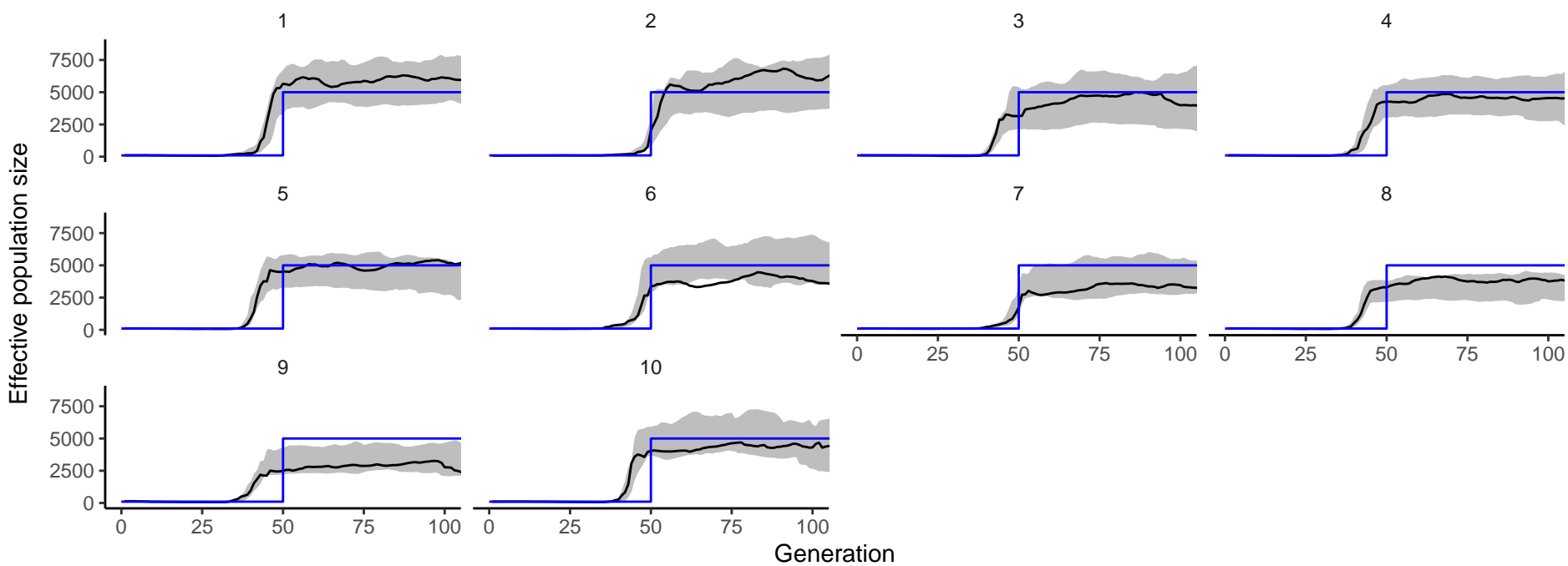

Leave one chromosome out

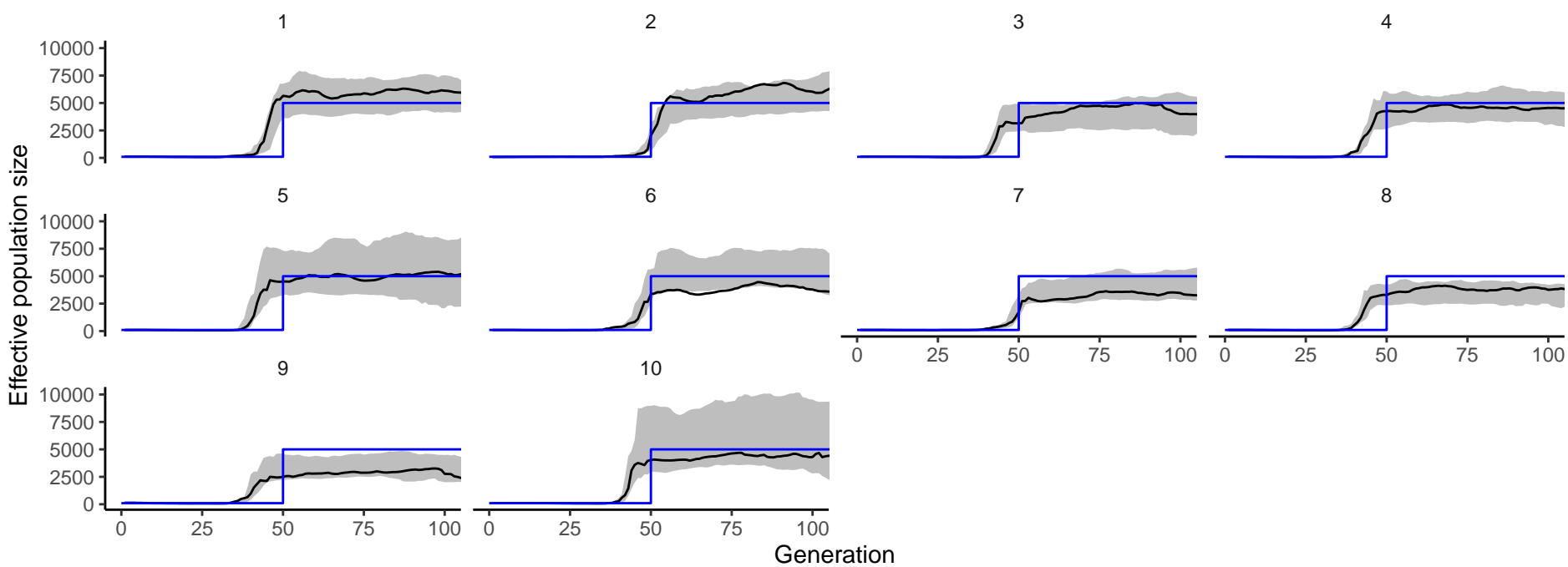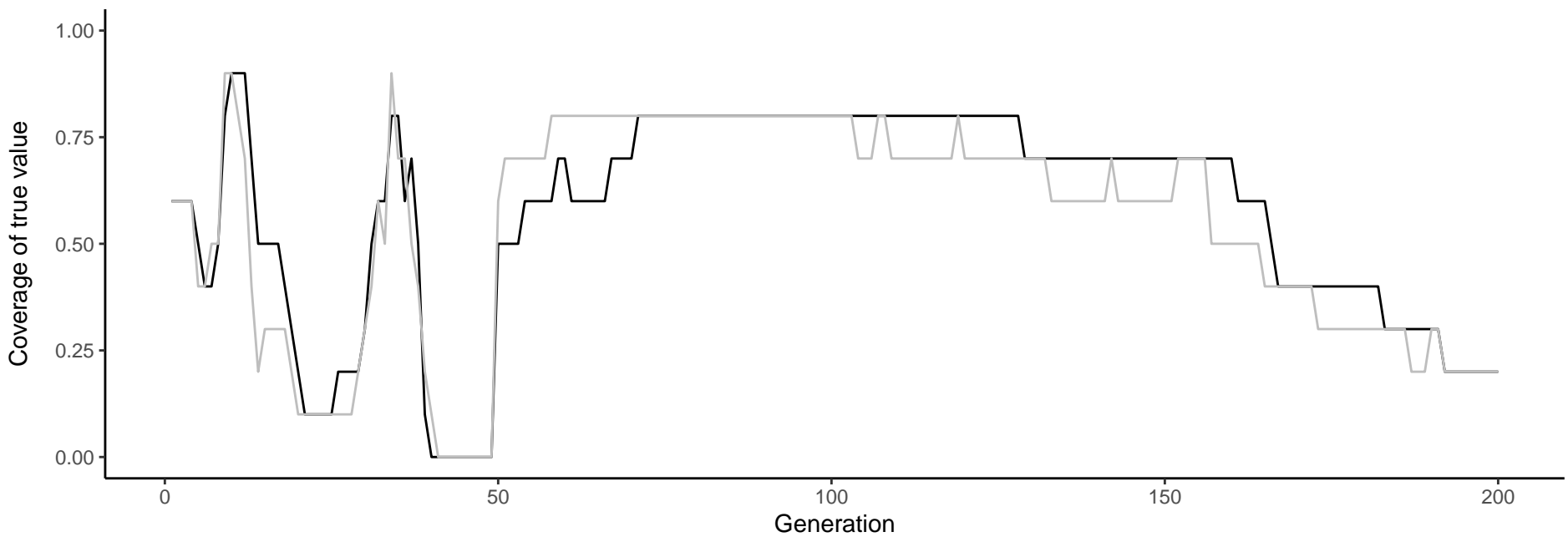

— Leave one chromosome out — Leave one sample out

Supplement: Supplementary file 2 — Additional file 2: Fig. 2. Testing of uncertainty intervals for GONE estimates. The panels show uncertainty intervals for 10 replicates of the simple decline simulation, constructed either by leaving one chromosome out or leaving one sample out. The black line shows the estimate from the full data. The blue line shows the true simulated population history. The bottom panel shows the average coverage of the intervals across generations, i.e., the fraction of replicates where the interval covers the true simulated value in that generation. [file 12711_2026_1050_MOESM2_ESM.pdf]

A

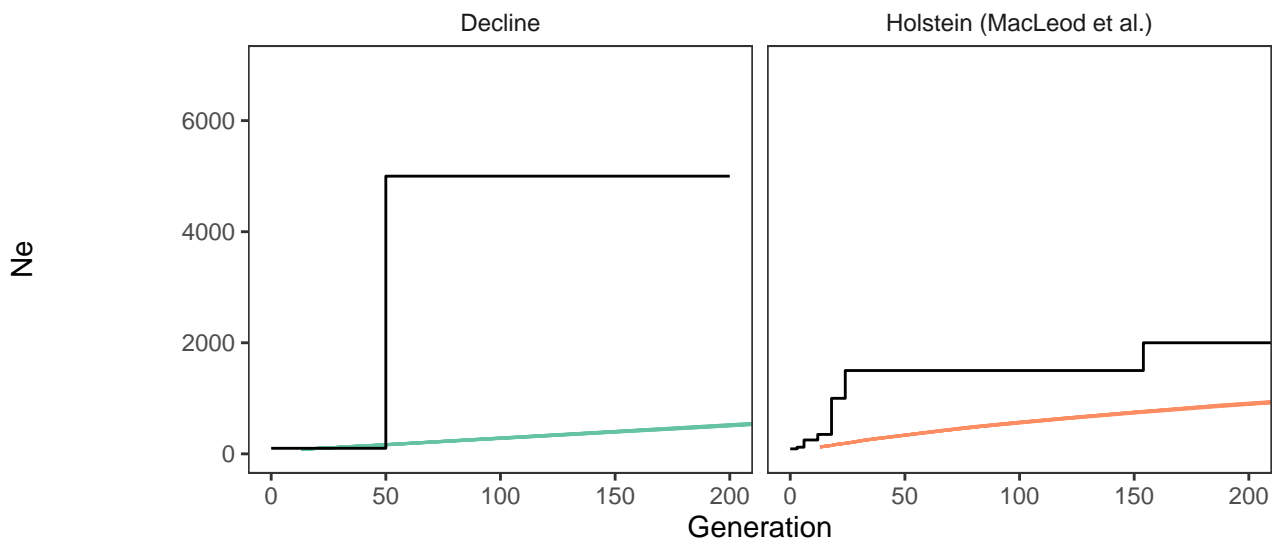

B

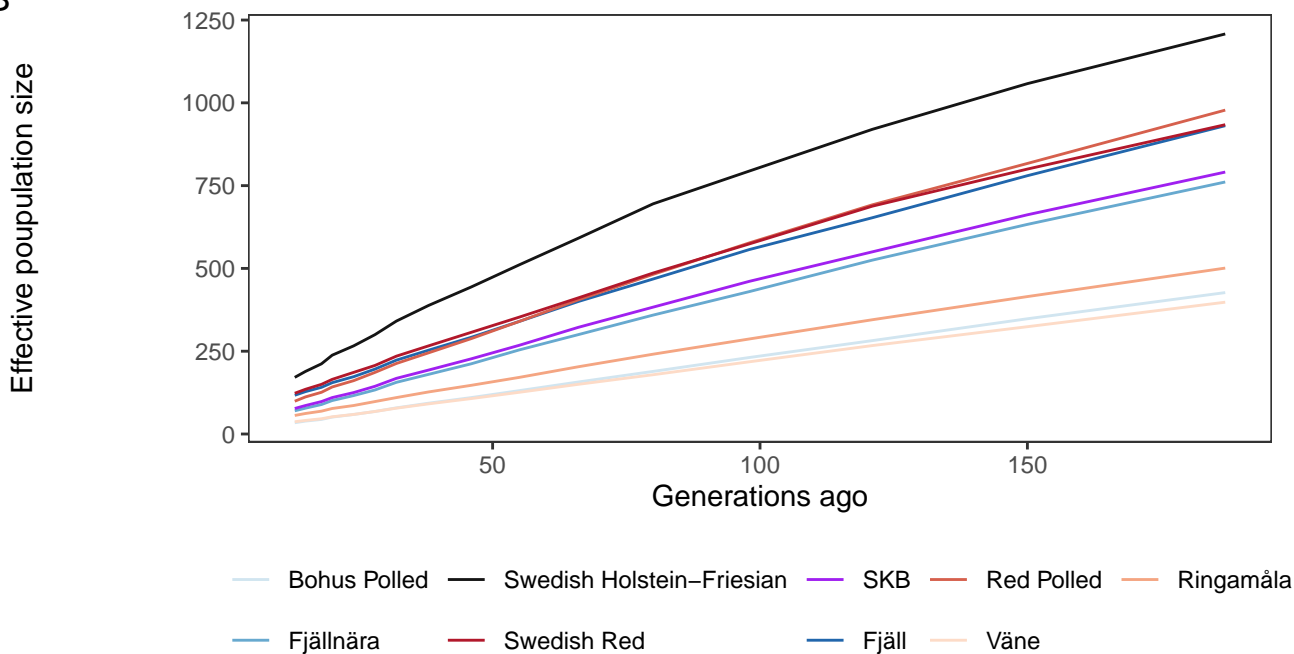

C

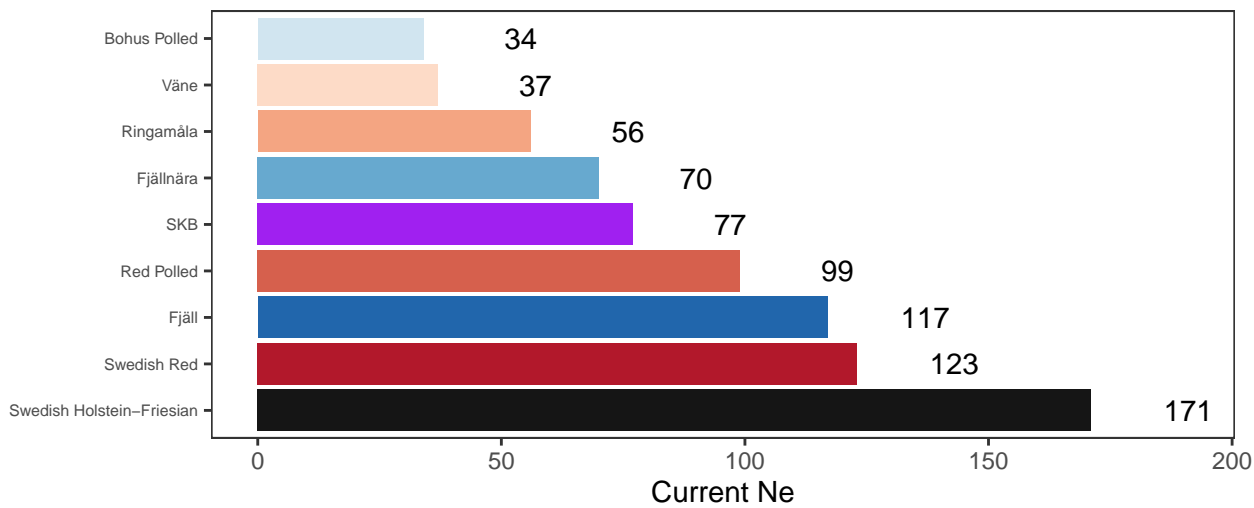

Supplement: Supplementary file 3 — Additional file3: Fig. S3. Evaluation of population history inference with SNeP. A) Population history inference with SNeP applied to simulated data. The black linesshow the true values of the simulated population histories. The coloured points show estimatedpopulation histories from 10 simulation replicates each. The simulated scenarios are simplepopulation histories with one (“Decline”) and a published population history for Holstein cattle(“Holstein MacLeod et al.”). B) Population histories of Swedish cattle breeds, estimated by SNePfrom SNP chip data. C) The bars show the estimated current population history (i.e., from the mostrecent generation) of Swedish cattle breeds with SNP chip data estimated by SNeP. [file 12711_2026_1050_MOESM3_ESM.pdf]

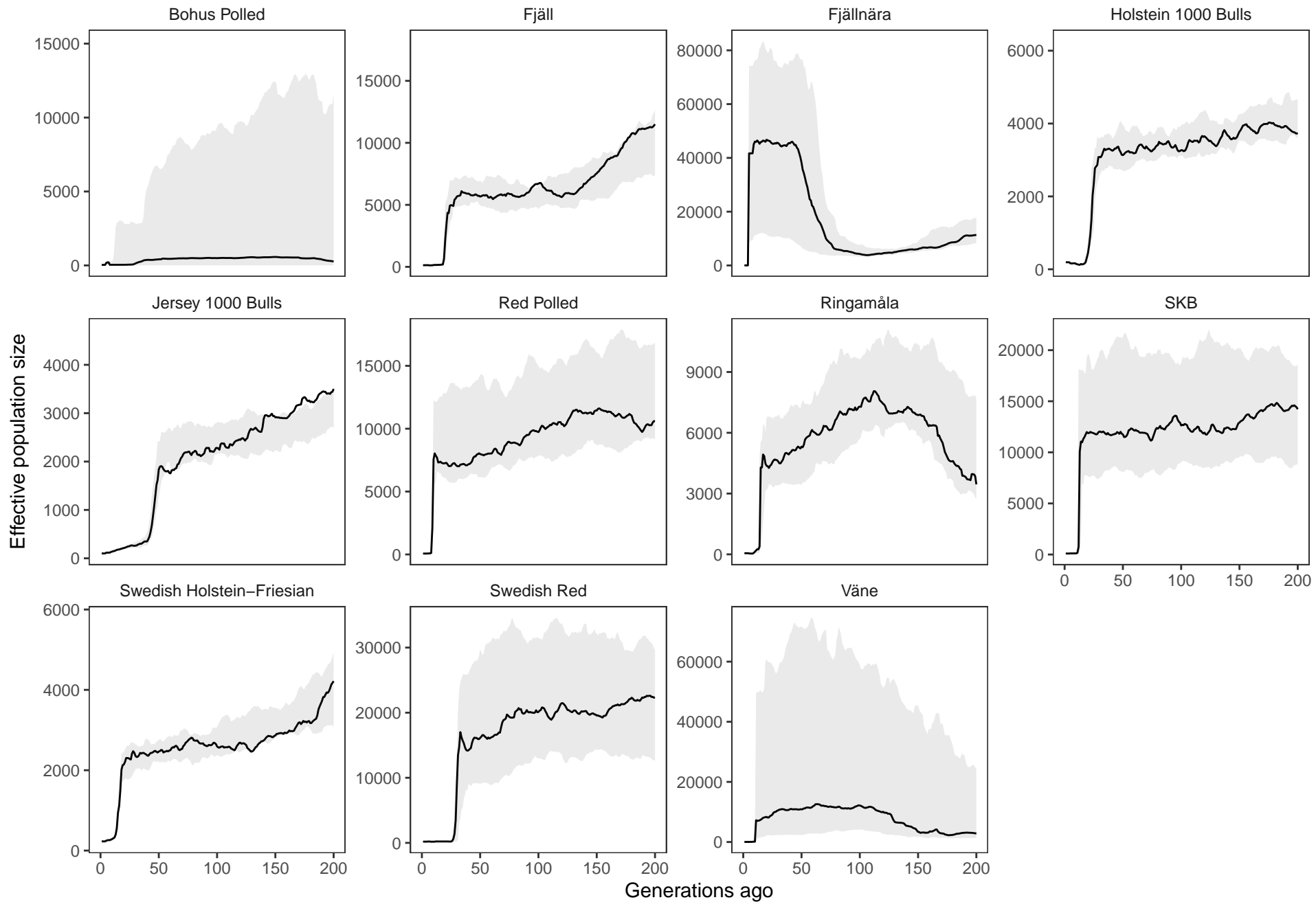

Supplement: Supplementary file 4 — Additional file4: Fig. S4. Population histories estimated by GONE. The horizontal axis shows time in generations, running backwards. The vertical axisshows the estimated effective population size. The shaded areas show uncertainty intervals made bydropping one sample at a time. This figure includes all estimated trajectories on their own scale. [file 12711_2026_1050_MOESM4_ESM.pdf]
