# Supplementary material for: Recent population history in Swedish cattle breeds
Source: Genet Sel Evol. 2026 May 24;58:27. doi: 10.1186/s12711-026-01050-z (PMC13202900; doi:10.1186/s12711-026-01050-z)
Supplement: Supplementary file 5 — Additional file5: Table S1. Summaries of estimated population histories. Time of the decline, estimated as the generation with the biggest decline in effectivepopulation size from the previous generation, and the mean effective population size before thedecline, from population histories estimated by GONE. [file 12711_2026_1050_MOESM5_ESM.pdf]

Table S1. Time of the decline, estimated as the generation with the biggest decline in effective population size from the previous generation, and the mean effective population size before the decline, from population histories estimated by GONE.

Breed	Time of decline (generation)	Mean size before decline
Fjäll	19	7,349
Red Polled	9	10,108
Ringamåla	14	6,291
SKB	12	12,870
Swedish Holstein-Friesian	17	2,852
Swedish Red	29	20,021
Väne	10	7,698
Holstein 1000 Bulls	23	3,586
Jersey 1000 Bulls	47	2,608
